# Reasons for referral to a social prescribing program through the COVID-19 pandemic and associated factors: a cross-sectional study in Portugal

**DOI:** 10.3389/fpubh.2026.1726269

**Published:** 2026-05-26

**Authors:** Louíse Viecili Hoffmeister, Vasco Ricoca Peixoto, Ana Gama, Pedro Aguiar, Sónia Dias

**Affiliations:** 1 NOVA National School of Public Health, ENSP, Public Health Research Centre, Comprehensive Health Research Center, CHRC, REAL, CCAL, NOVA University Lisbon, Lisbon, Portugal; 2 Department of Social Responsibility, Hospital Moinhos de Vento, Porto Alegre, Rio Grande do Sul, Brazil

**Keywords:** social prescribing, primary healthcare, reason for referral, patient profile, health promotion

## Abstract

**Introduction:**

Social prescribing (SP) links patients with non-medical needs affecting their health to services and activities within the community as a strategy to prevent disease and promote health and wellbeing. There is limited evidence on the reasons for referral to SP through time and of factors associated with specific reasons of referral. Understanding SP referral patterns is relevant to inform practice and policy. We analyzed factors associated with specific reasons for referral to SP program in Lisbon, including the differences through time.

**Methods:**

We conducted a cross-sectional study on referrals to a SP program between September 2018 and December 2023 in Lisbon. Data was collected through SP referrals forms. Reasons for referral and patient characteristics were described and compared in three periods (pre, during and Post-COVID-19 pandemic). Poisson regressions with robust estimation were used to identify factors associated with main reasons for referral including patient characteristics, comorbidities, and co-existing reasons.

**Results:**

Overall, 1,298 referrals were made to SP. Reasons for referral were Social and financial support (58.9%), Social Isolation (29.3%), Unemployment (19.8%), Mental health concerns (18.8%), Functional dependency (16.0%), Sedentary lifestyle (13.0%). Strong associations were found between Social Isolation, Mental Health concerns, and sedentary lifestyle as co-existing reasons for referral. These reasons were less common among patients referred for Functional dependency and Social and Financial support. We found a significant reduction in the proportion of sedentary lifestyle as reason for referral in the Post-COVID-19 period when compared to the Pre-COVID-19, but an increase in the proportion of Cardiovascular Disease, Obesity and Diabetes.

**Conclusions:**

Our findings suggest that community services and activities that broadly respond to social isolation, mental health, and sedentary lifestyle as commonly associated reasons for referral should be promoted. Changes in reasons for referral to SP through time reflect the influence of social dynamics on populations needs and should be monitored to broaden SP preventive and health promoting potential.

## 1 Introduction

Social prescribing (SP) is an innovative, intersectoral integrated care model that has garnered significant attention to its potential to address both health and social care needs. By linking patients with non-medical needs affecting their health to sources of support within the community, SP offers a holistic approach to healthcare (1). The SP model is founded on the understanding that the population health status is influenced not only by medical factors but also deeply intertwined with social, economic, and environmental determinants (2). This wide range of factors, also named as social determinants of health (SDOH), includes education access, healthcare access, neighborhood and built environment, social context, and economic stability (3, 4). WHO publications highlight that various risk factors significantly increase the likelihood of developing diseases or injuries. These include nutritional, behavioral, metabolic, environmental factors (5).

Research has shown that SDOH play a crucial role in health equity, influencing outcomes in various conditions such as heart failure, cancer, traumatic injuries, diabetes, and adverse pregnancy outcomes (6–10). Addressing SDOH through interventions has been linked to improved health outcomes (11, 12), as integrating information about these determinants into patient care plans has shown to lead to better health results (13). However, implementing these strategies can be complex and challenging. Healthcare providers and services often face constrains as limited time, insufficient resources, and uncertainty about how to effectively address patients' social needs (14–16). Conversely, some professionals believe that addressing SDOH will lead to greater job satisfaction, an enhanced perception of healthcare quality, and a deeper understanding of their patients' needs (13).

These findings reinforce the relevance of implementing interventions like SP, which provide complementary solutions to delivery of health and social integrated care. Based on a recent Delphi study conducted with a multidisciplinary panel of experts, SP is considered as “a method for trusted individuals in clinical and community settings to identify that a person has non-medical, health-related social needs and to subsequently connect them to non-clinical supports and services within the community by co-producing a social prescription—a non-medical prescription, to improve health and well- being and to strengthen community connections” (17). The co-production of SP is carried out collaboratively by the link worker and the patient. The link worker plays a crucial role in SP, acting as a bridge between healthcare services and community-based support. The community services often include, but are not limited to, art classes, group learning, gardening, befriending, cooking classes, healthy eating advice, and a variety of sports (1, 18).

The reasons for referring a patient to SP are multifaceted, reflecting the complexity of social determinants that influence health. The characteristics of patients referred to SP can be different depending on the context where the SP program is implemented. Evidence indicates that varying reasons for referral to SP are linked to distinct sociodemographic profiles and health profiles, highlighting the importance of understanding referral patterns to better tailor interventions e improve program effectiveness (19).

The COVID-19 pandemic was a period that significantly impacted the delivery of health and social care services worldwide (20, 21). In the context of SP, the limited available evidence suggests that, considering the pandemic's effects on health and living conditions, there were notable changes in patients' non-clinical needs (22). During the COVID-19 pandemic, the reasons for referral shifted away from the original eligibility criteria in many SP initiatives, with link workers focusing on providing immediate assistance and referrals to other services such as food and medicine supply deliveries (23). They also played a pivotal role in ensuring that people understood government guidelines and were coping with the challenges brought on by the pandemic (23). The lives of SP patients who were doing well prior to lockdown may subsequently deteriorated, prompting link workers to become creative and innovative in how they supported them through remote service delivery (23).

While SP is inherently shaped by broader SDOH, including socioeconomic conditions, such dimensions are not directly captured within routine referral data systems. Therefore, this study adopts a pragmatic approach by examining referral patterns and associated characteristics as documented in clinical practice, offering insight into how social needs are operationalized within SP programs. Moreover, although follow-up and outcome evaluation are central to the broader assessment of SP interventions, the present study is specifically designed to explore referral dynamics rather than patient trajectories or intervention effectiveness.

Although some studies have described reasons for referral to SP programs, few have explored the factors associated with each reason of referral, the relationships between these reasons and how these factors changed over pandemic period. By identifying referral profiles and dynamics, this study may help inform the design of joint actions and strategies aimed to address multiple needs simultaneously such as SP, thereby enhancing the efficiency and effectiveness of similar initiatives in times of social instability like the pandemic.

In this context, the aim of this study was to analyze the factors associated with specific reasons for referral to a SP program in Lisbon, including the impact across the periods before, during and after the COVID-19 pandemic.

## 2 Material and methods

### 2.1 Study setting and intervention

The study was conducted in two Family Healthcare Units (FHUs) located in downtown Lisbon, Portugal. These units deliver general healthcare to a population of approximately 30,000 individuals, spanning all age groups within their designated areas of responsibility (24, 25). The users of these units include individuals in vulnerable situations, such as older adults in isolation, recently arrived migrants, unemployed adults with low levels of education, and people experiencing homelessness.

This was the first SP program in Portugal and was launched in September 2018 within these FHUs using a bottom-up implementation approach. The program aims to enhance the health and wellbeing of the population enrolled in the FHUs by fostering collaboration among health services, social services, community organizations, and the voluntary sector.

Overall, patients of all age groups with non-clinical needs, who are assisted in these units, can be referred to the SP program by their GP, family nurses, or psychologists. Additionally, patients may self-refer to the program. During the appointment, the health professional may identify non-clinical needs and then explain the SP service to the patient, inquiring about their interests in being referred to an SP appointment. If the patient consents, the health professional completes a referral form using a computer-based platform. Following the consultation with the health professional, the patient is directed to the service counter in order to schedule an SP appointment with the link worker.

In this SP program, the role of the link worker in SP is carried out by social workers based at the FHUs, who are responsible for receiving referrals to the SP service. While link workers in this program are trained social workers, their role extends beyond traditional social work functions in primary care by emphasizing the co-production of individualized activity plans, active linkage with community-based resources, and structured follow-up to support patient engagement with non-clinical interventions.

During the SP appointment, the link worker develops an individual care plan with the patient using a participatory and collaborative approach, exploring with the patients their specific needs, context, motivations and interests, and jointly identifying the most suitable responses available through the service and community partners. The available responses include Senior University classes, Portuguese classes for migrants, support with medication, food and housing assistance, aqua aerobics and other types of physical activity, day centers, trishaw rides, among others. Generally, these responses are provided to patients free of charge.

After the referral process and the SP appointment, the link worker may continue to contact the patient to follow their pathway and, if necessary, schedule further appointments. The link worker may also reach out to health professionals who referred the patient and community services, to exchange information regarding the patient's adherence to the care plan and their level of satisfaction.

### 2.2 Study design and participants

A cross-sectional study was carried out to analyze the factors associated with specific reasons for referral to SP program in Lisbon during September 2018 and December 2023.

### 2.3 Data collection tools and techniques

Data were collected using the SP referral forms and electronic records from FHUs. The referral form is completed by the professional who refers the patient and includes the date of the referral, the reasons for referral (presented as a pre-defined list of 16 reasons), and patient's chronic diseases. Patient's sex and age were collected directly from electronic records. Self-referrals were not recorded through the referral form and, therefore, were not included in the sample analyzed. Data collection was carried out continuously throughout the project implementation by researchers.

### 2.4 Variables

The variables considered in this study were related to reasons for referral, sex, age, presence of chronic diseases and referral period.

The variable “reasons for referral” was used as a dependent variable. The original predefined list of 16 referral reasons included in the referral form comprised: social isolation; migrant integration; mental health; sedentary lifestyle/access to physical activity; social support/lack of awareness of social and health benefits; financial difficulties/indebtedness; support with medication purchase/treatment adherence; unemployment/labor issues/training; nutrition; housing; functional dependency; substance use (drugs/alcohol/other addictions); homelessness; social risk behavior; social support in terminal situations or bereavement; and other reasons. These were grouped into broader analytical categories for the purposes of this study.

Reasons for referral were reorganized into six groups: (1) social and financial support, covering social and health benefits, migrant integration, financial difficulties or indebtedness, support in purchasing medicines, food, and housing support, homelessness, substance use (drugs/alcohol/other addictions), social risk behavior, social support in terminal situations or bereavement; and other reasons; (2) social isolation, including loneliness; (3) unemployment, including other labor issues or the need for training or education; (4) mental health, including anxiety, depression and sadness; (5) functional dependency, involving the need for assistance to perform daily activities due to physical, cognitive, or emotional limitations; and (6) sedentary lifestyle, involving the need or willingness to undertake physical activity.

Sociodemographic data included sex, classified as “female” or “male”, and age calculated through the difference in years between date of birth and date of referral. The age data was then categorized in four groups: ≤ 25 years, 26–50 years, 51–75 years and >75 years.

Chronic diseases were categorized into groups, namely: (i) cardiovascular diseases – hypertension, coronary heart disease, heart failure; (ii) cerebrovascular diseases – stroke, dementia, other neurological diseases; (iii) obesity, overweight, and associated metabolic diseases; (iv) diabetes – also includes pre-diabetes; (v) respiratory diseases – chronic obstructive pulmonary disease, asthma, other respiratory diseases; (vi) mental illness – depression, anxiety, other mental illness; (vii) osteoarticular diseases – arthritis, osteoporosis, other osteoarticular disease; (viii) Oncological diseases; and (ix) Chronic pain.

The referral period was captured from the information on the day of referral. Day of referral was categorized by month and year and then grouped into three periods: Pre-COVID-19 (from 28/09/2018, the date of the first referral to the SP program, to 13/03/2020, when exceptional and temporary measures were established in Portugal in response to the COVID-19 epidemiological situation); during the COVID-19 (14/03/2020–21/04/2022 - when some of the restrictive measures in force were revised, such as the removal of the obligation to wear face masks in enclosed spaces); and Post-COVID-19, the period after the end of contingency state until the end of this study (22/04/2022–31/12/2023).

### 2.5 Data analysis

The unit of observation was the referral to SP, with each referral being assigned a unique ID. The fact that SP referral was chosen as unit of analysis rather than the patient referred minimizes loss of information regarding different referrals made to the same patient. Patients referred more than once were treated as new referrals if the referrals were made for different reasons. All patient referrals were checked for duplicates through patient health number. When a duplicated referral was identified (i.e. referral made for the same reason, regardless of whether made in the same day or in different days), the case was excluded from analysis to avoid overrepresentation of unchanged needs and to ensure independence between observations.

Data from the SP referral forms and FHUs electronic records were exported to an SPSS database (pseudonymized data). Descriptive statistics were employed. The proportion of each specific reason for referral was calculated by each patient's characteristics and other reasons for referral. To adjust for confounding, we used fully adjusted multivariable models using Poisson regression with robust estimation for binomial outcomes and calculated adjusted prevalence ratios with a 95% confidence interval. All independent variables were included in the Poisson models and no stepwise regression techniques were applied.

We analyzed the proportion of patient characteristics and reasons for referral overall and in the three periods: pre-pandemic, during pandemic, and post-pandemic. A chi-square independence test was performed to compare proportions of referral characteristics in the three periods (sex, age, reasons of referral and chronic diseases), using a significance level of 5%. All statistical analyzes were performed using Stata (version 14).

### 2.6 Ethics approval

This study is part of the “Evaluation of the Social Prescribing project in Health Units in the Lisbon and Tagus Valley region” project approved by the Ethics Committee for Health of the Regional Health Administration of Lisbon and Tagus Valley (reference 5 2020/CES/2020). Informed consent was not required, as the study was based on anonymized secondary data and involved no direct interaction with participants, in accordance with ethical guidelines.

All data handling and information-sharing procedures complied with national regulations and the General Data Protection Regulation (GDPR). At the time of referral, patients provided consent for the healthcare professional to share relevant information with the link worker as part of the SP process. Data were pseudonymized for research purposes, and only the minimum necessary information was shared between healthcare professionals, link workers, and community organizations. Communication followed secure institutional protocols to ensure confidentiality and data protection.

## 3 Results

During the study period, 1,298 referrals were made to SP program. As shown in Table 1, 64.8% (*n* = 841) of the referrals were female participants. Overall, the majority of referrals involved participants aged 51–75 years (33.4%, *n* = 433), followed by those aged over 75 years (27.6%, *n* = 358) and 26–50 years (27.2%, *n* = 353). The reasons for referral among participants included the need for social and financial support, noted in 58.9% (*n* = 765) of participants; social isolation, affecting 29.3% (*n* = 380) of individuals; unemployment, impacting 19.8% (*n* = 257) of individuals; mental health issues, identified in 18.8% (*n* = 244) of participants; and functional dependency, reported by 16.0% (*n* = 208) of participants. Additionally, 13.0% (*n* = 169) of participants were referred due to a sedentary lifestyle. The prevalence of chronic diseases within the sample is notable. Cardiovascular diseases affected 36.3% (*n* = 471) of participants, cerebrovascular disease was present in 14.9% (*n* = 194) of individuals, and obesity, overweight, and associated metabolic disorders were observed in 22.2% (*n* = 288) of participants. Additionally, other chronic conditions included diabetes, affecting 15.7% (*n* = 204) of individuals; respiratory diseases, reported by 8.9% (*n* = 116) of participants; mental illness, affecting 28.0% (*n* = 363) of participants; osteoarticular diseases, present in 16.9% (*n* = 219) of individuals; oncological diseases, affecting 4.7% (*n* = 61) of participants; and chronic pain, reported by 5.4% (*n* = 70) of individuals. 5.6% (*n* = 73) of patients were referred more than once.

**Table 1:** Characterization of referrals to the SP program in Lisbon, September 2018 to December 2023 (*n* = 1,298).

Variables	Category	Total sample (*n* = 1,298)
		*n (%)*
Sex	Female	841 (64.8)
	Male	457 (35.2)
Age	0–25	154 (11.9)
	26–50	353 (27.2)
	51–75	433 (33.4)
	>75	358 (27.6)
Reasons of referral	Social isolation	380 (29.3)
	Sedentary lifestyle	169 (13.0)
	Mental health	244 (18.8)
	Unemployment	257 (19.8)
	Social and financial support	765 (58.9)
	Functional dependency	208 (16.0)
Chronic diseases	Cardiovascular diseases	471 (36.3)
	Cerebrovascular disease	194 (14.9)
	Metabolic diseases	288 (22.2)
	Diabetes	204 (15.7)
	Respiratory diseases	116 (8.9)
	Mental illness	363 (28.0)
	Osteoarticular diseases	219 (16.9)
	Oncological diseases	61 (4.7)
	Chronic pain	70 (5.4)

### 3.1 Factors associated with specific reasons for referral

Referral for social isolation emerged as a strong predictor of other referral reasons. Individuals referred due to isolation had an increased likelihood of also being referred for sedentary behavior (aPR = 1.84, CI: [1.30–2.60], *p* = 0.001; aPR = 1.27, CI: [1.05–1.52], *p* = 0.013) and mental health concerns (aPR = 3.62, CI: [2.77–4.73], *p* < 0.001; aPR = 2.30, CI: [1.93–2.75], *p* < 0.001). Similarly, referrals for sedentary lifestyle were significantly related to mental health referrals, (aPR = 1.55, CI: [1.20–2.01], *p* = 0.001; aPR = 1.75, CI: [1.25–2.45], *p* = 0.001).

By contrast, referrals due to functional dependency tended to occur less frequently among sedentary behavior referrals (aPR = 0.51, CI: [0.28–0.91], *p* = 0.024), and individuals referred for mental health concerns were less likely to be referred for functional dependency (aPR = 0.51, CI: [0.33–0.76], *p* = 0.001).

Significant associations also involved social and financial support needs. Individuals referred for this reason were less likely to have referrals for social isolation (aPR = 0.51, CI: [0.43–0.61], *p* < 0.001), sedentary behavior (aPR = 0.47, CI: [0.34–0.66], *p* < 0.001), mental health issues (aPR = 0.70, CI: [0.55–0.89], *p* = 0.003), and functional dependency (aPR = 0.73, CI: [0.58–0.92], *p* = 0.008). These associations were generally consistent in both directions, suggesting that different types of vulnerability tend to manifest through distinct and sometimes mutually exclusive referral patterns.

Furthermore, referrals due to functional dependency were associated with a significantly lower likelihood of unemployment referrals (aPR = 0.17, CI: [0.04–0.69], *p* = 0.013).

### 3.2 Referral reasons and associations with sex, age and chronic diseases

The associations between referral reasons and sex, age, and chronic diseases were presented for the most frequent referral reasons: social and financial support, social isolation, unemployment and functional dependency. Regarding referrals due to social and financial support (Table 2), individuals aged 26–50 and 51–75 were more likely to be referred to SP for this support compared to those aged 0–25, with aPR of 1.23 (95% CI: 1.05–1.45, *p* = 0.012) and 1.48 (95% CI: 1.26–1.74, *p* < 0.001), respectively. Individuals with cardiovascular diseases were less likely to be referred for social and financial support (aPR = 0.87, 95% CI: 0.79–0.97, *p* = 0.012), while those with cerebrovascular diseases were more likely to be referred for it (aPR = 1.15, 95% CI: 1.01–1.30, *p* = 0.030). Respiratory diseases and osteoarticular diseases were linked to higher referrals of support (aPR = 1.24, 95% CI: 1.10–1.39, *p* < 0.001; aPR = 1.22, 95% CI: 1.10–1.36, *p* < 0.001, respectively). The proportion of patients referred to SP for this reason did not differ across sexes.

**Table 2:** Association between demographic factors, other reasons of referral, chronic diseases and “Social and financial support” as reason of referral to the SP program, Lisbon, Portugal.

Variables	Category	Total	Social and financial support	SocFi*n*%	aPR	CI95%	*p*
Demographic factors							
Sex	Female	841	480	57.07			Ref
	Male	457	285	62.36	0.98	[0.90–1.07]	0.641
Age	0–25	154	82	53.25			Ref
	26–50	353	221	62.61	**1.23**	[1.05–1.45]	0.012^*^
	51–75	433	296	68.36	**1.48**	[1.26–1.74]	< 0.001^**^
	>75	358	166	46.37	1.09	[0.89–1.34]	0.388
Other reasons of referral							
Social isolation	No	918	630	68.63			Ref
	Yes	380	135	35.53	**0.61**	[0.52–0.71]	< 0.001^**^
Sedentary lifestyle	No	1129	711	62.98			Ref
	Yes	169	54	31.95	**0.61**	[0.48–0.77]	< 0.001^**^
Mental health	No	1,054	670	63.57			Ref
	Yes	244	95	38.93	**0.80**	[0.67–0.95]	0.010^*^
Functional dependency	No	1,090	655	60.09			Ref
	Yes	208	110	52.88	**0.80**	[0.68–0.94]	0.007^*^
Unemployment	No	1,041	602	57.83			Ref
	Yes	257	163	63.42	0.99	[0.89–1.11]	0.902
Chronic diseases							
Cardiovascular diseases	No	827	503	60.82			Ref
	Yes	471	262	55.63	**0.87**	[0.79–0.97]	0.012^*^
Cerebrovascular disease	No	1,104	650	58.88			Ref
	Yes	194	115	59.28	**1.15**	[1.01–1.30]	0.030^*^
Obesity, overweight, and associated metabolic diseases	No	1,010	599	59.31			Ref
	Yes	288	166	57.64	1.00	[0.90–1.12]	0.943
Diabetes	No	1,094	638	58.32			Ref
	Yes	204	127	62.25	1.07	[0.95–1.20]	0.268
Respiratory diseases	No	1,182	679	57.45			Ref
	Yes	116	86	74.14	**1.24**	[1.10–1.39]	< 0.001^**^
Mental illness	No	935	568	60.75			Ref
	Yes	363	197	54.27	0.99	[0.90–1.10]	0.900
Osteoarticular diseases	No	1,079	626	58.02			Ref
	Yes	219	139	63.47	**1.22**	[1.10–1.36]	< 0.001^**^
Oncological diseases	No	1,237	727	58.77			Ref
	Yes	61	38	62.30	1.03	[0.86–1.23]	0.779
Chronic pain	No	1,228	724	58.96			Ref
	Yes	70	41	58.57	1.10	[0.90–1.35]	0.336

September 2018–December 2023 (*n* = 1,298).

SocFin%, proportion of the variable within the group referred by social and financial support; CI95%, confidence interval (95%); aPR, adjusted prevalence ratio; Ref, reference category.

The adjusted prevalence ratios shown in bold are statistically significant (*p* < 0.05). ^*^Values less than 0.05. ^**^Values equal to or less than 0.001.

Related to social isolation referrals, as presented in Table 3, male participants had a lower likelihood to be referred for social isolation compared to females (aPR = 0.73; CI95% [0.61–0.87]; *p* = 0.001). Participants aged 26–50 were more likely to be referred for social isolation compared to those younger, with an adjusted prevalence ratio (aPR) of 1.96 (CI: [1.13–3.38], *p* = 0.016). Participants aged 51–75 had a substantially higher likelihood of being referred for social isolation (aPR = 3.86, CI: [2.27–6.57], *p* < 0.001) and those aged over 75 had the highest likelihood (aPR = 5.60, CI: [3.26–9.61], *p* < 0.001). No significant association was found between referral for social isolation and chronic conditions.

**Table 3:** Association between demographic factors, other reasons of referral, chronic diseases and “Social isolation” as reason of referral to the SP program, Lisbon, Portugal.

Variables	Category	Total	Social isolation	Isol%	aPR	CI95%	*p*
Demographic factors							
Sex	Female	841	279	33.17			Ref
	Male	457	101	22.10	**0.73**	[0.61–0.87]	0.001^**^
Age	0–25	154	12	7.79			Ref
	26–50	353	56	15.86	**1.96**	[1.13–3.38]	0.016^*^
	51–75	433	142	32.79	**3.86**	[2.27–6.57]	< 0.001^**^
	>75	358	170	47.49	**5.60**	[3.26–9.61]	< 0.001^**^
Other reasons of referral							
Social and financial support	No	533	245	45.97			Ref
	Yes	765	135	17.65	**0.51**	[0.43–0.61]	< 0.001^**^
Sedentary lifestyle	No	1,129	280	24.80			Ref
	Yes	169	100	59.17	**1.27**	[1.05–1.52]	0.013^*^
Mental health	No	1,054	222	21.06			Ref
	Yes	244	158	64.75	**2.30**	[1.93–2.75]	< 0.001^**^
Functional dependency	No	1,090	320	29.36			Ref
	Yes	208	60	28.85	**0.73**	[0.57–0.93]	0.012^*^
Unemployment	No	1,041	338	32.47			Ref
	Yes	257	42	16.34	0.88	[0.66–1.17]	0.380
Chronic diseases							
Cardiovascular diseases	No	827	203	24.55			Ref
	Yes	471	177	37.58	0.96	[0.81–1.13]	0.628
Cerebrovascular disease	No	1,104	312	28.26			Ref
	Yes	194	68	35.05	1.04	[0.84–1.30]	0.700
Obesity, overweight, and associated metabolic diseases	No	1,010	279	27.62			Ref
	Yes	288	101	35.07	0.86	[0.72–1.04]	0.115
Diabetes	No	1,094	311	28.43			Ref
	Yes	204	69	33.82	1.18	[0.97–1.42]	0.096
Respiratory diseases	No	1,182	342	28.93			Ref
	Yes	116	38	32.76	1.12	[0.88–1.44]	0.356
Mental illness	No	935	230	24.60			Ref
	Yes	363	150	41.32	1.03	[0.87–1.23]	0.702
Osteoarticular diseases	No	1,079	295	27.34			Ref
	Yes	219	85	38.81	1.02	[0.86–1.22]	0.802
Oncological diseases	No	1,237	358	28.94			Ref
	Yes	61	22	36.07	1.22	[0.90–1.64]	0.198
Chronic pain	No	1,228	356	28.99			Ref
	Yes	70	24	34.29	0.98	[0.72–1.34]	0.913

September 2018–December 2023 (*n* = 1298).

Isol%, proportion of the variable within the group referred by social isolation; CI95%, confidence interval (95%); aPR, adjusted prevalence ratio; Ref, Reference category.

The adjusted prevalence ratios shown in bold are statistically significant (*p* < 0.05). ^*^Values less than 0.05. ^**^Values equal to or less than 0.001.

Related to functional dependency as reason for referral (Table 4), older age groups showed a significant association with being referred for functional dependency. Those aged 51–75 having an adjusted prevalence ratio (aPR) of 5.48 (95% CI: 1.33–22.57, *p* = 0.019), and those over 75 having an aPR of 15.76 (95% CI: 3.91–63.52, p < 0.001) compared to the youngest group ( ≤ 25 years). Additionally, having cerebrovascular disease (aPR = 2.57, 95% CI: 2.06–3.20, *p* < 0.001), diabetes (aPR = 1.43, 95% CI: 1.14–1.79, *p* = 0.002), and osteoarticular diseases (aPR = 1.41, 95% CI: 1.11–1.78, *p* = 0.005) significantly increased the likelihood of being referred due to functional dependency.

**Table 4:** Association between demographic factors, other reasons of referral, chronic diseases and “Functional dependency” as reason of referral to the SP program, Lisbon, Portugal.

Variables	Category	Total	Functional dependency	Func*t*%	aPR	CI95%	*p*
Demographic factors							
Sex	Female	841	134	15.93			Ref
	Male	457	74	16.19	1.14	[0.93–1.41]	0.212
Age	0–25	154	2	1.30			Ref
	26–50	353	11	3.12	2.78	[0.63–12.33]	0.179
	51–75	433	42	9.70	**5.48**	[1.33–22.57]	0.019^*^
	>75	358	153	42.74	**15.76**	[3.91–63.52]	< 0.001^**^
Other reasons of referral							
Social and financial support	No	533	98	18.39			Ref
	Yes	765	110	14.38	0.73	[0.58–0.92]	0.008^*^
Social isolation	No	918	148	16.12			Ref
	Yes	380	60	15.79	**0.65**	[0.51–0.84]	0.001^**^
Sedentary lifestyle	No	1,129	197	17.45			Ref
	Yes	169	11	6.51	**0.51**	[0.28–0.91]	0.024^*^
Mental health	No	1,054	188	17.84			Ref
	Yes	244	20	8.20	**0.58**	[0.39–0.88]	0.011^*^
Unemployment	No	1,041	206	19.79			Ref
	Yes	257	2	0.78	**0.13**	[0.03–0.54]	0.005^*^
Chronic diseases							
Cardiovascular diseases	No	827	88	10.64			Ref
	Yes	471	120	25.48	0.94	[0.74–1.18]	0.577
Cerebrovascular disease	No	1,104	107	9.69			Ref
	Yes	194	101	52.06	**2.57**	[2.06–3.20]	< 0.001^**^
Obesity, overweight, and associated metabolic diseases	No	1,010	148	14.65			Ref
	Yes	288	60	20.83	0.98	[0.76–1.27]	0.881
Diabetes	No	1,094	151	13.80			Ref
	Yes	204	57	27.94	**1.43**	[1.14–1.79]	0.002^*^
Respiratory diseases	No	1,182	180	15.23			Ref
	Yes	116	28	24.14	0.84	[0.59–1.20]	0.341
Mental illness	No	935	140	14.97			Ref
	Yes	363	68	18.73	1.26	[0.99–1.60]	0.061
Osteoarticular diseases	No	1,079	147	13.62			Ref
	Yes	219	61	27.85	**1.41**	[1.11–1.78]	0.005^*^
Oncological diseases	No	1,237	189	15.28			Ref
	Yes	61	19	31.15	1.53	[0.98–2.37]	0.060
Chronic pain	No	1,228	188	15.31			Ref
	Yes	70	20	28.57	1.38	[0.94–2.01]	0.097

September 2018–December 2023 (*n* = 1,298).

Funct%, proportion of the variable within the group referred by functional dependency; CI95%, confidence interval (95%); aPR, adjusted prevalence ratio; Ref, reference category.

The adjusted prevalence ratios shown in bold are statistically significant (*p* < 0.05). ^*^Values less than 0.05. ^**^Values equal to or less than 0.001.

Regarding the factors associated with unemployment as a reason for referral (Table 5), males had a higher likelihood of being referred due to unemployment compared to females (aPR = 1.38, 95% CI: [1.14–1.69], *p* = 0.001). Age was also a significant factor, with individuals aged 26–50 being more likely to be referred by unemployment than those aged 0–25 (aPR = 1.51, 95% CI: [1.12–2.04], *p* = 0.008), while those aged 51–75 were less likely to be referred due to unemployment (aPR = 0.63, 95% CI: [0.42–0.94], *p* = 0.024). Obesity, overweight, and associated metabolic diseases were associated with a higher likelihood of to be referred due to unemployment (aPR = 1.45, 95% CI: [1.09–1.93], *p* = 0.011).

**Table 5:** Association between demographic factors, other reasons of referral, chronic diseases and “Unemployment” as reason of referral to the SP program, Lisbon, Portugal.

Variables	Category	Total	Unemployment	Unemp%	aPR	CI95%	*p*
Demographic factors							
Sex	Female	841	143	17.00			Ref
	Male	457	114	24.95	**1.38**	[1,14–1,69]	0,001^**^
Age	0–25	154	42	27.27			Ref
	26–50	353	149	42.21	**1.51**	[1,12–2,04]	0,008^*^
	51–75	433	66	15.24	**0.63**	[0,42–0,94]	0,024^*^
	>75	358	0	0.00	**0.00**	[0,00–0,00]	0,001^**^
Other reasons of referral							
Social and financial support	No	533	94	17.64			Ref
	Yes	765	163	21.31	0.99	[0,79–1,25]	0.931
Social isolation	No	918	215	23.42			Ref
	Yes	380	42	11.05	0.87	[0,63–1,19]	0,374
Sedentary lifestyle	No	1.129	233	20.64			Ref
	Yes	169	24	14.20	0.70	[0,47–1,06]	0,089
Mental health	No	1.054	203	19.26			Ref
	Yes	244	54	22.13	1.24	[0,92–1,67]	0,158
Functional dependency	No	1,090	255	23.39			Ref
	Yes	208	2	0.96	**0.17**	[0,04–0,69]	0,013^*^
Chronic diseases							
Cardiovascular diseases	No	827	208	25.15			Ref
	Yes	471	49	10.40	1.05	[0,77–1,44]	0,757
Cerebrovascular disease	No	1,104	247	22.37			Ref
	Yes	194	10	5.15	0.70	[0,39–1,25]	0,230
Obesity, overweight, and associated metabolic diseases	No	1,010	208	20.59			Ref
	Yes	288	49	17.01	**1.45**	[1,09–1,93]	0,011^*^
Diabetes	No	1,094	231	21.12			Ref
	Yes	204	26	12.75	0.82	[0,58–1,16]	0,256
Respiratory diseases	No	1,182	247	20.90			Ref
	Yes	116	10	8.62	0.64	[0,35–1,16]	0,142
Mental illness	No	935	195	20.86			Ref
	Yes	363	62	17.08	0.93	[0,70–1,22]	0,584
Osteoarticular diseases	No	1,079	229	21.22			Ref
	Yes	219	28	12.79	1.19	[0,86–1,66]	0,289
Oncological diseases	No	1,237	254	20.53			Ref
	Yes	61	3	4.92	0.41	[0,14–1,26]	0,120
Chronic pain	No	1,228	246	20.03			Ref
	Yes	70	11	15.71	1.10	[0,70–1,72]	0,688

September 2018–December 2023 (*n* = 1298).

Unemp%, proportion of the variable within the group referred by unemployment; CI95%, confidence interval (95%); aPR, adjusted prevalence ratio; Ref, reference category.

The adjusted prevalence ratios shown in bold are statistically significant (*p* < 0.05). ^*^Values less than 0.05. ^**^Values equal to or less than 0.001.

Other reasons for referral and the corresponding associated factors are reported in the tables provided in the Supplementary Material 1.

### 3.3 Changes in referral reasons across pre-, during, and Post-COVID-19 periods

**Figure 1:**
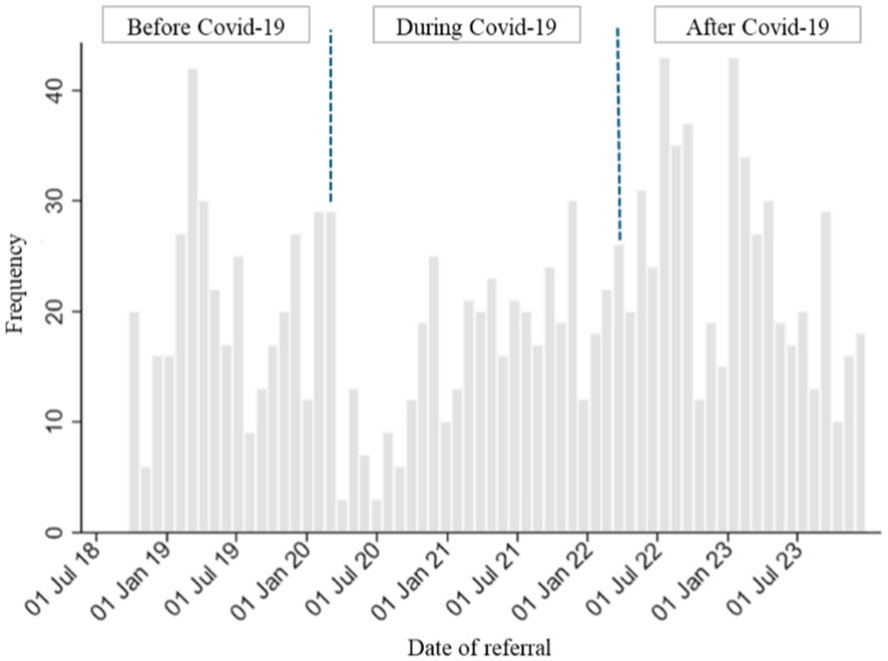
Distribution of referrals to SP program in Lisbon from September 2018 to December 2023 by month and COVID-19 periods.

Regarding the period of referral, 28.9% (*n* = 375) occurred before the pandemic period, 32.4% (*n* = 421) took place during the pandemic period and 38.7% (*n* = 502) were made after the pandemic period (Figure 1).

Before the COVID-19 pandemic, SP program in Lisbon had an average of 10.5 (±5.1) referrals per month, which rose to 33.7 referrals/month; *p* < 0.001 during the pandemic and rose further in the post-pandemic period, 53.2 referrals/month. Demographic characteristics of those referenced was stable across periods with higher frequency of woman and older people. Social and financial support was the leading reason for referral across periods, although with different frequencies (Pre-COVID-19: 49.9%; during COVID-19: 68.2%; Post-COVID-19: 58.0%). The same patterns were observed for chronic conditions with cardiovascular diseases and mental illness being the most frequent across the three periods with minor differences. The table with detailed results can be consulted in Supplementary Material 2.

The analysis revealed significant changes in the distribution of reasons for referral to the SP program across the analyzed periods as shown in proportion of referrals with a specific reason (Table 6). Referrals for social and financial support increased its proportion significantly during the COVID-19 period, considering all referrals that were done in that period (*p* < 0.001). Social isolation, which had been one of the most prevalent reasons for referral prior to the pandemic, showed a significant decline in relative proportion during (*p* < 0.001) Referrals for sedentary lifestyle decreased it's proportion markedly during (*p* < 0.001) and after the pandemic (*p* < 0.001). Functional dependency showed the most pronounced increase in proportion during the pandemic, with referrals more than doubling in proportion (9.33 vs. 22.57%) compared to the pre-pandemic period (*p* < 0.001), remaining statistically higher after COVID-19 (*p* = 0.007). Lastly, proportion of referrals with unemployment as reason remained stable across all three periods.

**Table 6:** Proportions of each reason for referral to the SP programme among all referrals by period and *p*-value of the difference in proportions, Lisbon, Portugal.

Reason of referral	Referral period	*n* (%)	*p*
Social and financial support	Before COVID-19	187 (49.87)	Ref
	During COVID-19	287 (68.17)	< 0.001^**^
	After COVID-19	291 (57.97)	0.017^*^
Social isolation	Before COVID-19	140 (37.33)	Ref
	During COVID-19	99 (23.52)	< 0.001^**^
	After COVID-19	141 (28.09)	0.004
Sedentary lifestyle	Before COVID-19	90 (24.00)	Ref
	During COVID-19	30 (7.13)	< 0.001^**^
	After COVID-19	49 (9.76)	< 0.001^**^
Mental health	Before COVID-19	81 (21.60)	Ref
	During COVID-19	80 (19.00)	0.362
	After COVID-19	83 (16.53)	0.057
Functional dependency	Before COVID-19	35 (9.33)	Ref
	During COVID-19	95 (22.57)	< 0.001^**^
	After COVID-19	78 (15.54)	0.007^*^
Unemployment	Before COVID-19	71 (18.93)	Ref
	During COVID-19	93 (22.09)	0.272
	After COVID-19	93 (18.53)	0.878

September 2018–December 2023 (*n* = 1298).

Ref, reference category. ^*^Values less than 0.05. ^**^Values equal to or less than 0.001.

## 4 Discussion

Our study enabled the identification of the main reasons for referral to SP in Lisbon, along with the factors associated with each reason and associations between them while examining variation in referral counts and proportion of referrals with specific reasons across the periods before, during, and after COVID-19 pandemic. The most commons reasons for referral were Social and financial support (58.9%), Social Isolation (29.3%), Unemployment (19.8%), Mental health concerns (18.8%), Functional dependency (16.0%), Sedentary lifestyle (13.0%). Strong associations were found between Social Isolation, Mental Health concerns, and sedentary lifestyle as co-existing reasons for referral.

Considering the analytical findings regarding factors associated with main reasons for referral, we can hypothesize 4 patient profiles in the context of SP in the study context: (a) Patients referred due to needs of Social and financial support from 50 to 75, irrespective of sex, without coexisting reasons for referral to SP; (b) Patients referred due to a combination of Social isolation, mental health concerns and sedentary lifestyle, more often female and of older age irrespective of comorbidities; (c) Patients referred due to Functional Dependency, of older age groups, irrespective of sex, without coexisting reasons of referral and with cerebrovascular disease, diabetes and osteoarticular disease; and (d) Patients referred for unemployment, more commonly males, from 26 to 50 years old, without other reasons for referral and irrespective of comorbidities. These profiles should be interpreted cautiously, as they are derived from cross-sectional associations and do not represent longitudinal patient trajectories or causal pathways. They allow for generating hypothesis and regarding referral profiles.

Among the referrals in the study period a higher proportion of female referrals were identified (64.8%), with the largest proportion being of patients aged between 51 and 75 years (33.4%) in terms of age groups. In the FHUs where the program is implemented, female users represent approximately 51% of the population (*n* = 14,549; *N* = 28,548). Users aged 75 or older account for 8.3%, and the dependency ratio is 53.3% (26, 27). A cross-sectional study conducted in Spain between September 2018 and March 2021, which analyzed 2,109 electronic health records in primary healthcare centers about sociodemographic and SP protocol data, reported a similar pattern (28). SP was more frequently applied to older females, with significant sex differences observed (28). A study conducted in the west of Newcastle found that patients of pension age were most frequently referred for issues related to social isolation, mental health concerns and long-term condition management (29). Typically, referrals are made for individuals experiencing low mood, depression, anxiety, and social isolation, or those suffering from long-term conditions and the socio-economic consequences of poor health (29).

In this study, the most frequent reason for referral was social and financial support, as identified in other studies (30). This reason is believed to attract more attention and is often prioritized by referring professionals, as it addresses essential daily living needs. These include support for food, housing, the purchase of medicines, orthoses and prostheses, as well as assistance in assessing social security needs and benefits, such as medical certificates and tax exemptions. These needs directly impact patients' health and the ability to manage health effectively, making them a priority for healthcare professionals and patients (31, 32). Factors associated with social and financial support as reason for referral suggest that this is often a stand-alone reason of referral as demonstrated by the negative association or no association with other reasons of referral.

In our study, the second most frequent reason for referral was social isolation, including loneliness, which aligns with findings from other SP studies (33, 34). Although social isolation and loneliness are distinct concepts, they can co-occur. Social isolation is an objective measure related to the lack of social contact while loneliness is a subjective experience related to a context of social connectedness (35). Social isolation and loneliness are growing public health and public policy concerns, a prominence that has been amplified by the COVID-19 pandemic (36, 37). In our study, social isolation was strongly associated with older age, sedentary lifestyle, and mental health concerns as coexisting reasons for referral. This highlights a strong cluster of needs that should be considered when designing SP programs and promoting community responses.

In addition to addressing patients‘ socioeconomic needs, SP seeks to act more broadly, focusing on disease prevention and health promotion, fostering positive patient outcomes (38–40). This includes offering advice and support on lifestyle changes (e.g., physical activities, food habits), fostering community engagement, employment and training, promoting mental health, and delivering personalized emotional and social support (41). This is in line with our findings in terms of the prevalence of referrals for social isolation, sedentary lifestyle and mental health concerns among all referrals. This “out of the box” approach of SP requires a cultural shift among healthcare professionals, a change that is believed to be ongoing within these FHUs in Lisbon. Many activities and services in the communities may have a positive impact in social isolation, sedentary lifestyle and mental health and they should be prioritized to patients that combine these reasons for referrals.

Findings from our study on factors associated with SP referrals highlight a common profile for individuals referred due to social isolation. Key factors such as being female, older age, and sedentary behavior and mental health issues, as well as cardiovascular disease are associated with referral for social isolation reason. Women, as well as older individuals, are more vulnerable to feelings of loneliness, which significantly affects mental health and makes it more challenging for patients to maintain healthy habits (42).

Chronic diseases were also associated with various reasons for SP referrals, as it provides an opportunity to extend multidisciplinary care. SP serves as an effective tool to promote lifestyle change, engage individuals facing loneliness or isolation, and address unmet needs that may worsen chronic health conditions (43). Our findings align with the existing literature, regarding the association of specific reasons of referral with chronic diseases, namely cardiovascular, cerebrovascular, mental health, osteoarticular conditions and diabetes. (44, 45).

This study also analyzed changes in the proportion of reasons for referral to the SP program across three distinct periods: before, during, and after the COVID-19 pandemic. Changes are challenging to explain but may be attributed to variations in risk perception among healthcare professionals and patients, greater awareness of the SP program among referrers for certain conditions, changing expectations of healthcare professionals regarding services and activities that can address patients' needs and of the health impact of specific SP activities and services regarding patients' health needs. On the other hand, the healthcare system's overload during the pandemic, which may have driven GPs and FHUs to seek alternatives for complex cases (46).

Before the pandemic, referrals were largely driven by social isolation and sedentary lifestyle, consistent with SP's emphasis on prevention and promotion of social wellbeing (47). During the pandemic, the relative proportion of referrals changed. There was an increase in proportion of referrals for social and financial support and functional dependency, likely reflecting the prioritization of services to address socioeconomic hardship, disruption of services (48). Proportion of referrals for patients with partial or complete functional dependence have increased during the pandemic, possibly driven by the mobility restrictions imposed during this period (49, 50). Nonetheless, naturally all proportions presented represent a relative importance among all referrals in that period, as reductions in referrals for specific reasons may affect the proportion of other among all referrals, even considering coexistence of reasons of referral in the same referral. In the post-pandemic period, there was an increase in the prevalence of chronic conditions in referrals for SP– particularly cardiovascular disease, diabetes, and obesity. This finding may be understood in light of reports of reduced healthcare access, delays in diagnosis, and worsening of existing conditions during the pandemic (51–53) or related to prioritization of SP for patients with chronic conditions. These changes should be interpreted cautiously, as they may reflect not only shifts in population needs but also adaptations in healthcare delivery, referral practices, and service availability during the COVID-19 pandemic.

### 4.1 Implications for practice and policy

The clustering of social isolation, mental health concerns, and sedentary lifestyle highlights the importance of SP referrals to activities and services that address these co-occurring needs. Social isolation, sedentary lifestyle and mental health concerns form a group of important risk factors, both influencing the likelihood of each other and of other negative health outcomes. These findings suggest that interventions combining social engagement, physical activity promotion, and mental health support may be particularly relevant in this context, especially when designed to respond to overlapping and interdependent needs.

From a practice perspective, these results underscore the potential value of refining referral guidance to better capture complex patient profiles and support more tailored and coordinated responses. This may include strengthening the training of prescribers and link workers to improve the identification of co-existing needs and to facilitate more comprehensive care planning. In addition, enhancing coordination between healthcare services and community-based organizations appears to be essential for ensuring continuity of care and optimizing patient engagement with prescribed interventions.

From a policy perspective, the findings point to the relevance of supporting the development and scaling of community-based resources capable of addressing multiple needs simultaneously, rather than single-need interventions. This may contribute to a more efficient allocation of resources and to the strengthening of SP as a preventive and health-promoting strategy. Monitoring changes in referral patterns over time may also provide useful insights to inform planning and prioritization within SP programs.

Finally, from a research perspective, further studies are needed to better understand how different referral profiles translate into specific intervention pathways, as well as to assess the effectiveness and implementation of integrated SP approaches in addressing complex social needs.

### 4.2 Limitations

This study presents insights into social prescribing referrals; however, limitations should be considered. Firstly, the sample is drawn from two FHUs in Lisbon, which may not be representative of the broader Portuguese population or of SP practices in other contexts. As the SP program was implemented in a specific regional setting, the findings are influenced by local population characteristics, healthcare professionals' practices, and referral behaviors.

Second, the study relies on healthcare professionals' assessments recorded in referral forms, which may introduce reporting bias. Professionals may prioritize certain patient needs over others based on perceptions of expected benefit, awareness of available community services, or institutional constraints.

Although SP was closely linked to SDOH, this study did not include direct socioeconomic indicators such as income, education, or deprivation indices. This limitation should be considered when interpreting the social vulnerability profiles identified, as these were inferred from referral reasons rather than directly measured socioeconomic conditions. Furthermore, this study focused on referral patterns and associated factors and did not assess patient follow-up, adherence, or outcomes after referral.

In addition, the unit of analysis was the referral rather than the individual. Although only a small proportion of patients had multiple referrals, referrals from the same individual may increase strength if specific associations are overrepresented in this group. Furthermore, self-referrals of patients were possible in this program but were not included in the dataset. Our focus in this analysis remains on referrals from healthcare professionals.

Finally, the cross-sectional design precludes causal inference. Temporal comparisons across COVID-19 periods should also be interpreted with caution, as observed changes may reflect not only shifts in population needs but also system-level adaptations in healthcare delivery, referral practices, healthcare accessibility, and provider awareness. Future research should explore how referral reasons and patients characteristics are associated with specific activities and services prescribed and adherence, and explore longitudinal analyzes to further validate trends and assess the long-term impact of SP interventions on patient behaviors and health outcomes.

## 5 Conclusion

This study provides valuable insights into the evolving patterns of SP referrals in a program in Lisbon, particularly across pre-, during, and Post-COVID-19 pandemic periods. Our findings highlight social and financial support emerging as the most common reason for referral, especially during the pandemic, possibly reflecting the socioeconomic disruptions experienced by vulnerable populations. Additionally, social isolation, mental health, and sedentary lifestyle formed a group of strongly associated referral reasons that frequently co-occur, underscoring the interconnected nature of psychosocial and behavioral determinants of health and suggesting the need to support and increase community solutions that can simultaneously address these health needs while promoting healthcare professionals' attention to these relevant risk factors and awareness of potential options within SP. In contrast, functional dependency appeared as a standalone reason for referral, particularly among older patients with chronic conditions such as diabetes, cerebrovascular diseases, and osteoarticular disorders, reinforcing the urgency of providing comprehensive care for individuals with significant limitations in autonomy. The observed post-pandemic proportional increase in referrals of patients with chronic disease, including cardiovascular disease, obesity, and diabetes, suggest a role of SP in addressing unmet needs for chronic conditions beyond the clinical approach, reinforcing its capacity to bridge gaps in traditional healthcare models. Moving forward, efforts should focus on refining referral guidance for different needs, ensuring equitable access to SP services, and adapting interventions, particularly in response to the ongoing impacts of social isolation and lifestyle-related conditions. Additionally, given the decline in sedentary lifestyle and social isolation-related referrals, strategies should maintain awareness among health professionals regarding these important health risk factors as well as the preventive potential of referrals to SP, ensuring that preventive interventions regain momentum post-pandemic. By continuously monitoring referral trends, policymakers and practitioners can optimize SP initiatives, fostering effective, and responsive community-based healthcare models. Facilitating communication among new projects that investigate referral patterns is necessary to promote integrated solutions for main reasons for referral and to inform practice and policy in SP.

## Data Availability

The datasets presented in this article are not readily available because the dataset used in this study contains sensitive participant information and is therefore not publicly available. Access may be granted upon reasonable request to the corresponding author, in accordance with ethical and institutional regulations. Requests to access the datasets should be directed to sonia.dias@ensp.unl.pt.
