# Supplementary material for: Reasons for referral to a social prescribing program through the COVID-19 pandemic and associated factors: a cross-sectional study in Portugal
Source: Front Public Health. 2026 May 26;14:1726269. doi: 10.3389/fpubh.2026.1726269 (PMC13246674; doi:10.3389/fpubh.2026.1726269)
Supplement: Supplementary file 1 [file Table_1.docx]

**Supplementary Material 1.** Tables presenting the associations between demographic factors, chronic diseases, and all reasons for referral.

**Table 1.** Association between demographic factors, other reasons of referral, chronic diseases and “Social and financial support” as reason of referral to the SP program, Lisbon, Portugal. September 2018 - December 2023 (n=1298).

| **Variables** | **Category** | **Total** | **Social and financial support** | **SocFin%** | **crudePR** | **CI95%** | ***p*** | **aPR** | **CI95%** | ***p*** |
| --- | --- | --- | --- | --- | --- | --- | --- | --- | --- | --- |
| **Demographic factors** |  |  |  |  |  |  |  |  |  |  |
| Sex | Female | 841 | 480 | 57.07 | Ref | Ref | Ref | Ref | Ref | Ref |
|  | Male | 457 | 285 | 62.36 | 1.09 | [1.00-1.20] | 0.064 | 0.98 | [0.90-1.07] | 0.641 |
| Age | 0-25 | 154 | 82 | 53.25 | Ref | Ref | Ref | Ref | Ref | Ref |
|  | 26-50 | 353 | 221 | 62.61 | 1.18 | [0.99-1.39] | 0.048 | 1.23 | [1.05-1.45] | 0.012 |
|  | 51-75 | 433 | 296 | 68.36 | 1.28 | [1.09-1.51] | 0.001 | 1.48 | [1.26-1.74] | <0.001 |
|  | >75 | 358 | 166 | 46.37 | 0.87 | [0.72-1.05] | 0.153 | 1.09 | [0.89-1.34] | 0.388 |
| **Other reasons of referral** |  |  |  |  |  |  |  |  |  |  |
| Social isolation | No | 918 | 630 | 68.63 | Ref | Ref | Ref | Ref | Ref | Ref |
|  | Yes | 380 | 135 | 35.53 | 0.52 | [0.45-0.60] | <0.001 | 0.61 | [0.52-0.71] | <0.001 |
| Sedentary lifestyle | No | 1129 | 711 | 62.98 | Ref | Ref | Ref | Ref | Ref | Ref |
|  | Yes | 169 | 54 | 31.95 | 0.51 | [0.41-0.64] | <0.001 | 0.61 | [0.48-0.77] | <0.001 |
| Mental health | No | 1054 | 670 | 63.57 | Ref | Ref | Ref | Ref | Ref | Ref |
|  | Yes | 244 | 95 | 38.93 | 0.61 | [0.52-0.72] | <0.001 | 0.80 | [0.67-0.95] | 0.010 |
| Functional dependency | No | 1090 | 655 | 60.09 | Ref | Ref | Ref | Ref | Ref | Ref |
|  | Yes | 208 | 110 | 52.88 | 0.88 | [0.77-1.01] | 0.053 | 0.80 | [0.68-0.94] | 0.007 |
| Unemployment | No | 1041 | 602 | 57.83 | Ref | Ref | Ref | Ref | Ref | Ref |
|  | Yes | 257 | 163 | 63.42 | 1.10 | [0.99-1.22] | 0.103 | 0.99 | [0.89-1.11] | 0.902 |
| **Chronic diseases** | |  |  |  |  |  |  |  |  |  |
| Cardiovascular diseases | No | 827 | 503 | 60.82 | Ref | Ref | Ref | Ref | Ref | Ref |
|  | Yes | 471 | 262 | 55.63 | 0.91 | [0.83-1.01] | 0.067 | 0.87 | [0.79-0.97] | 0.012 |
| Cerebrovascular disease | No | 1104 | 650 | 58.88 | Ref | Ref | Ref | Ref | Ref | Ref |
|  | Yes | 194 | 115 | 59.28 | 1.01 | [0.89-1.14] | 0.916 | 1.15 | [1.01-1.30] | 0.030 |
| Obesity, overweight, and associated metabolic diseases | No | 1010 | 599 | 59.31 | Ref | Ref | Ref | Ref | Ref | Ref |
|  | Yes | 288 | 166 | 57.64 | 0.97 | [0.87-1.09] | 0.612 | 1.00 | [0.90-1.12] | 0.943 |
| Diabetes | No | 1094 | 638 | 58.32 | Ref | Ref | Ref | Ref | Ref | Ref |
|  | Yes | 204 | 127 | 62.25 | 1.07 | [0.95-1.20] | 0.294 | 1.07 | [0.95-1.20] | 0.268 |
| Respiratory diseases | No | 1182 | 679 | 57.45 | Ref | Ref | Ref | Ref | Ref | Ref |
|  | Yes | 116 | 86 | 74.14 | 1.29 | [1.15-1.45] | <0.001 | 1.24 | [1.10-1.39] | <0.001 |
| Mental illness | No | 935 | 568 | 60.75 | Ref | Ref | Ref | Ref | Ref | Ref |
|  | Yes | 363 | 197 | 54.27 | 0.89 | [0.80-0.99] | 0.033 | 0.99 | [0.90-1.10] | 0.900 |
| Osteoarticular diseases | No | 1079 | 626 | 58.02 | Ref | Ref | Ref | Ref | Ref | Ref |
|  | Yes | 219 | 139 | 63.47 | 1.09 | [0.98-1.22] | 0.135 | 1.22 | [1.10-1.36] | <0.001 |
| Oncological diseases | No | 1237 | 727 | 58.77 | Ref | Ref | Ref | Ref | Ref | Ref |
|  | Yes | 61 | 38 | 62.30 | 1.06 | [0.87-1.30] | 0.585 | 1.03 | [0.86-1.23] | 0.779 |
| Chronic pain | No | 1228 | 724 | 58.96 | Ref | Ref | Ref | Ref | Ref | Ref |
|  | Yes | 70 | 41 | 58.57 | 0.99 | [0.81-1.22] | 0.949 | 1.10 | [0.90-1.35] | 0.336 |

**SocFin%**= proportion of the variable within the group referred by social and financial support; **crudePR**=Crude Prevalence Ratio; **CI95%**=Confidence Interval (95%); **aPR**=Adjusted Prevalence Ratio; **Ref**=Reference category.

**Table 2.** Association between demographic factors, other reasons of referral, chronic diseases and “Social Isolation” as reason of referral to the SP program, Lisbon, Portugal. September 2018 - December 2023 (n=1298).

| **Variables** | **Category** | **Total** | **Social isolation** | **Isol%** | **crudePR** | **CI95%** | ***p*** | **aPR** | **CI95%** | ***p*** |
| --- | --- | --- | --- | --- | --- | --- | --- | --- | --- | --- |
| **Demographic factors** |  |  |  |  |  |  |  |  |  |  |
| Sex | Female | 841 | 279 | 33.17 | Ref | Ref | Ref | Ref | Ref | Ref |
|  | Male | 457 | 101 | 22.10 | 0.67 | [0.55-0.81] | <0.001 | 0.73 | [0.61-0.87] | 0.001 |
| Age | 0-25 | 154 | 12 | 7.79 | Ref | Ref | Ref | Ref | Ref | Ref |
|  | 26-50 | 353 | 56 | 15.86 | 2.04 | [1.12-3.69] | 0.014 | 1.96 | [1.13-3.38] | 0.016 |
|  | 51-75 | 433 | 142 | 32.79 | 4.21 | [2.40-7.37] | <0.001 | 3.86 | [2.27-6.57] | <0.001 |
|  | >75 | 358 | 170 | 47.49 | 6.09 | [3.50-10.61] | <0.001 | 5.60 | [3.26-9.61] | <0.001 |
| **Other reasons of referral** |  |  |  |  |  |  |  |  |  |  |
| Social and financial support | No | 533 | 245 | 45.97 | Ref | Ref | Ref | Ref | Ref | Ref |
|  | Yes | 765 | 135 | 17.65 | 0.38 | [0.32-0.46] | <0.001 | 0.51 | [0.43-0.61] | <0.001 |
| Sedentary lifestyle | No | 1129 | 280 | 24.80 | Ref | Ref | Ref | Ref | Ref | Ref |
|  | Yes | 169 | 100 | 59.17 | 2.39 | [2.03-2.80] | <0.001 | 1.27 | [1.05-1.52] | 0.013 |
| Mental health | No | 1054 | 222 | 21.06 | Ref | Ref | Ref | Ref | Ref | Ref |
|  | Yes | 244 | 158 | 64.75 | 3.07 | [2.65-3.57] | <0.001 | 2.30 | [1.93-2.75] | <0.001 |
| Functional dependency | No | 1090 | 320 | 29.36 | Ref | Ref | Ref | Ref | Ref | Ref |
|  | Yes | 208 | 60 | 28.85 | 0.98 | [0.78-1.24] | 0.882 | 0.73 | [0.57-0.93] | 0.012 |
| Unemployment | No | 1041 | 338 | 32.47 | Ref | Ref | Ref | Ref | Ref | Ref |
|  | Yes | 257 | 42 | 16.34 | 0.50 | [0.38-0.67] | <0.001 | 0.88 | [0.66-1.17] | 0.380 |
| **Chronic diseases** | |  |  |  |  |  |  |  |  |  |
| Cardiovascular diseases | No | 827 | 203 | 24.55 | Ref | Ref | Ref | Ref | Ref | Ref |
|  | Yes | 471 | 177 | 37.58 | 1.53 | [1.30-1.81] | <0.001 | 0.96 | [0.81-1.13] | 0.628 |
| Cerebrovascular disease | No | 1104 | 312 | 28.26 | Ref | Ref | Ref | Ref | Ref | Ref |
|  | Yes | 194 | 68 | 35.05 | 1.24 | [1.00-1.54] | 0.055 | 1.04 | [0.84-1.30] | 0.700 |
| Obesity, overweight, and associated metabolic diseases | No | 1010 | 279 | 27.62 | Ref | Ref | Ref | Ref | Ref | Ref |
|  | Yes | 288 | 101 | 35.07 | 1.27 | [1.05-1.53] | 0.014 | 0.86 | [0.72-1.04] | 0.115 |
| Diabetes | No | 1094 | 311 | 28.43 | Ref | Ref | Ref | Ref | Ref | Ref |
|  | Yes | 204 | 69 | 33.82 | 1.19 | [0.96-1.47] | 0.120 | 1.18 | [0.97-1.42] | 0.096 |
| Respiratory diseases | No | 1182 | 342 | 28.93 | Ref | Ref | Ref | Ref | Ref | Ref |
|  | Yes | 116 | 38 | 32.76 | 1.13 | [0.86-1.49] | 0.388 | 1.12 | [0.88-1.44] | 0.356 |
| Mental illness | No | 935 | 230 | 24.60 | Ref | Ref | Ref | Ref | Ref | Ref |
|  | Yes | 363 | 150 | 41.32 | 1.68 | [1.42-1.98] | <0.001 | 1.03 | [0.87-1.23] | 0.702 |
| Osteoarticular diseases | No | 1079 | 295 | 27.34 | Ref | Ref | Ref | Ref | Ref | Ref |
|  | Yes | 219 | 85 | 38.81 | 1.42 | [1.17-1.72] | 0.001 | 1.02 | [0.86-1.22] | 0.802 |
| Oncological diseases | No | 1237 | 358 | 28.94 | Ref | Ref | Ref | Ref | Ref | Ref |
|  | Yes | 61 | 22 | 36.07 | 1.25 | [0.88-1.76] | 0.233 | 1.22 | [0.90-1.64] | 0.198 |
| Chronic pain | No | 1228 | 356 | 28.99 | Ref | Ref | Ref | Ref | Ref | Ref |
|  | Yes | 70 | 24 | 34.29 | 1.18 | [0.85-1.65] | 0.344 | 0.98 | [0.72-1.34] | 0.913 |

**Isol%=** proportion of the variable within the group referred by social isolation; **crudePR=**Crude Prevalence Ratio; **CI95%**=Confidence Interval (95%); **aPR**=Adjusted Prevalence Ratio; **Ref**=Reference category.

**Table 3.** Association between demographic factors, other reasons of referral, chronic diseases and “Sedentary lifestyle” as reason of referral to the SP program, Lisbon, Portugal. September 2018 - December 2023 (n=1298).

| **Variables** | **Category** | **Total** | **Sedentary lifestyle** | **Sedent%** | **crudePR** | **CI95%** | ***p*** | **aPR** | **CI95%** | ***p*** |
| --- | --- | --- | --- | --- | --- | --- | --- | --- | --- | --- |
| **Demographic factors** |  |  |  |  |  |  |  |  |  |  |
| Sex | Female | 841 | 125 | 14.86 | Ref | Ref | Ref | Ref | Ref | Ref |
|  | Male | 457 | 44 | 9.63 | 0.65 | [0.47-0.90] | 0.007 | 0.85 | [0.62-1.17] | 0.322 |
| Age | 0-25 | 154 | 9 | 5.84 | Ref | Ref | Ref | Ref | Ref | Ref |
|  | 26-50 | 353 | 35 | 9.92 | 1.70 | [0.84-3.44] | 0.134 | 1.53 | [0.78-3.02] | 0.216 |
|  | 51-75 | 433 | 83 | 19.17 | 3.28 | [1.69-6.36] | <0.001 | 2.20 | [1.12-4.32] | 0.022 |
|  | >75 | 358 | 42 | 11.73 | 2.01 | [1.00-4.02] | 0.041 | 1.36 | [0.64-2.88] | 0.426 |
| **Other reasons of referral** |  |  |  |  |  |  |  |  |  |  |
| Social and financial support | No | 533 | 115 | 21.58 | Ref | Ref | Ref | Ref | Ref | Ref |
|  | Yes | 765 | 54 | 7.06 | 0.33 | [0.24-0.44] | <0.001 | 0.47 | [0.34-0.66] | <0.001 |
| Social isolation | No | 918 | 69 | 7.52 | Ref | Ref | Ref | Ref | Ref | Ref |
|  | Yes | 380 | 100 | 26.32 | 3.50 | [2.64-4.64] | <0.001 | 1.84 | [1.30-2.60] | 0.001 |
| Mental health | No | 1054 | 97 | 9.20 | Ref | Ref | Ref | Ref | Ref | Ref |
|  | Yes | 244 | 72 | 29.51 | 3.21 | [2.44-4.21] | <0.001 | 1.75 | [1.25-2.45] | 0.001 |
| Functional dependency | No | 1090 | 158 | 14.50 | Ref | Ref | Ref | Ref | Ref | Ref |
|  | Yes | 208 | 11 | 5.29 | 0.36 | [0.20-0.66] | <0.001 | 0.45 | [0.23-0.89] | 0.020 |
| Unemployment | No | 1041 | 145 | 13.93 | Ref | Ref | Ref | Ref | Ref | Ref |
|  | Yes | 257 | 24 | 9.34 | 0.67 | [0.45-1.01] | 0.050 | 0.76 | [0.49-1.18] | 0.216 |
| **Chronic diseases** | |  |  |  |  |  |  |  |  |  |
| Cardiovascular diseases | No | 827 | 103 | 12.45 | Ref | Ref | Ref | Ref | Ref | Ref |
|  | Yes | 471 | 66 | 14.01 | 1.13 | [0.84-1.50] | 0.423 | 0.72 | [0.51-1.03] | 0.069 |
| Cerebrovascular disease | No | 1104 | 150 | 13.59 | Ref | Ref | Ref | Ref | Ref | Ref |
|  | Yes | 194 | 19 | 9.79 | 0.72 | [0.46-1.13] | 0.148 | 1.04 | [0.67-1.61] | 0.864 |
| Obesity, overweight, and associated metabolic diseases | No | 1010 | 101 | 10.00 | Ref | Ref | Ref | Ref | Ref | Ref |
|  | Yes | 288 | 68 | 23.61 | 2.36 | [1.79-3.12] | <0.001 | 2.46 | [1.83-3.31] | <0.001 |
| Diabetes | No | 1094 | 143 | 13.07 | Ref | Ref | Ref | Ref | Ref | Ref |
|  | Yes | 204 | 26 | 12.75 | 0.98 | [0.66-1.44] | 0.899 | 0.85 | [0.58-1.25] | 0.413 |
| Respiratory diseases | No | 1182 | 159 | 13.45 | Ref | Ref | Ref | Ref | Ref | Ref |
|  | Yes | 116 | 10 | 8.62 | 0.64 | [0.35-1.18] | 0.140 | 0.62 | [0.33-1.18] | 0.144 |
| Mental illness | No | 935 | 100 | 10.70 | Ref | Ref | Ref | Ref | Ref | Ref |
|  | Yes | 363 | 69 | 19.01 | 1.78 | [1.34-2.36] | <0.001 | 1.02 | [0.76-1.37] | 0.882 |
| Osteoarticular diseases | No | 1079 | 125 | 11.58 | Ref | Ref | Ref | Ref | Ref | Ref |
|  | Yes | 219 | 44 | 20.09 | 1.73 | [1.27-2.37] | 0.001 | 1.62 | [1.17-2.23] | 0.003 |
| Oncological diseases | No | 1237 | 162 | 13.10 | Ref | Ref | Ref | Ref | Ref | Ref |
|  | Yes | 61 | 7 | 11.48 | 0.88 | [0.43-1.79] | 0.713 | 0.85 | [0.46-1.58] | 0.611 |
| Chronic pain | No | 1228 | 151 | 12.30 | Ref | Ref | Ref | Ref | Ref | Ref |
|  | Yes | 70 | 18 | 25.71 | 2.09 | [1.37-3.20] | 0.001 | 2.01 | [1.36-2.98] | <0.001 |

**Sedent%=** proportion of the variable within the group referred by sedentary lifestyle; **crudePR=**Crude Prevalence Ratio; **CI95%**=Confidence Interval (95%); **aPR**=Adjusted Prevalence Ratio; **Ref**=Reference category.

**Table 4.** Association between demographic factors, other reasons of referral, chronic diseases and “Mental health” as reason of referral to the SP program, Lisbon, Portugal. September 2018 - December 2023 (n=1298).

| **Variables** | **Category** | **Total** | **Mental health** | **Mental%** | **crudePR** | **CI95%** | ***p*** | **aPR** | **CI95%** | ***p*** |
| --- | --- | --- | --- | --- | --- | --- | --- | --- | --- | --- |
| **Demographic factors** |  |  |  |  |  |  |  |  |  |  |
| Sex | Female | 841 | 163 | 19.38 | Ref | Ref | Ref | Ref | Ref | Ref |
|  | Male | 457 | 81 | 17.72 | 0.91 | [0.72-1.16] | 0.465 | 1.11 | [0.90-1.37] | 0.322 |
| Age | 0-25 | 154 | 15 | 9.74 | Ref | Ref | Ref | Ref | Ref | Ref |
|  | 26-50 | 353 | 74 | 20.96 | 2.15 | [1.28-3.63] | 0.002 | 1.56 | [0.99-2.45] | 0.054 |
|  | 51-75 | 433 | 89 | 20.55 | 2.11 | [1.26-3.53] | 0.003 | 1.06 | [0.66-1.71] | 0.798 |
|  | >75 | 358 | 66 | 18.44 | 1.89 | [1.12-3.21] | 0.013 | 1.00 | [0.59-1.68] | 0.992 |
| **Other reasons of referral** |  |  |  |  |  |  |  |  |  |  |
| Social and financial support | No | 533 | 149 | 27.95 | Ref | Ref | Ref | Ref | Ref | Ref |
|  | Yes | 765 | 95 | 12.42 | 0.44 | [0.35-0.56] | <0.001 | 0.70 | [0.55-0.89] | 0.003 |
| Social isolation | No | 918 | 86 | 9.37 | Ref | Ref | Ref | Ref | Ref | Ref |
|  | Yes | 380 | 158 | 41.58 | 4.44 | [3.51-5.61] | <0.001 | 3.62 | [2.77-4.73] | <0.001 |
| Sedentary lifestyle | No | 1129 | 172 | 15.23 | Ref | Ref | Ref | Ref | Ref | Ref |
|  | Yes | 169 | 72 | 42.60 | 2.80 | [2.24-3.49] | <0.001 | 1.55 | [1.20-2.01] | 0.001 |
| Functional dependency | No | 1090 | 224 | 20.55 | Ref | Ref | Ref | Ref | Ref | Ref |
|  | Yes | 208 | 20 | 9.62 | 0.47 | [0.30-0.72] | <0.001 | 0.51 | [0.33-0.76] | 0.001 |
| Unemployment | No | 1041 | 190 | 18.25 | Ref | Ref | Ref | Ref | Ref | Ref |
|  | Yes | 257 | 54 | 21.01 | 1.15 | [0.88-1.51] | 0.310 | 1.24 | [0.93-1.65] | 0.146 |
| **Chronic diseases** |  |  |  |  |  |  |  |  |  |  |
| Cardiovascular diseases | No | 827 | 156 | 18.86 | Ref | Ref | Ref | Ref | Ref | Ref |
|  | Yes | 471 | 88 | 18.68 | 0.99 | [0.78-1.25] | 0.936 | 0.80 | [0.62-1.05] | 0.103 |
| Cerebrovascular disease | No | 1104 | 211 | 19.11 | Ref | Ref | Ref | Ref | Ref | Ref |
|  | Yes | 194 | 33 | 17.01 | 0.89 | [0.64-1.24] | 0.490 | 1.10 | [0.82-1.47] | 0.544 |
| Obesity, overweight, and associated metabolic diseases | No | 1010 | 174 | 17.23 | Ref | Ref | Ref | Ref | Ref | Ref |
|  | Yes | 288 | 70 | 24.31 | 1.41 | [1.10-1.80] | 0.007 | 1.21 | [0.92-1.58] | 0.177 |
| Diabetes | No | 1094 | 217 | 19.84 | Ref | Ref | Ref | Ref | Ref | Ref |
|  | Yes | 204 | 27 | 13.24 | 0.67 | [0.46-0.97] | 0.027 | 0.72 | [0.51-1.01] | 0.059 |
| Respiratory diseases | No | 1182 | 221 | 18.70 | Ref | Ref | Ref | Ref | Ref | Ref |
|  | Yes | 116 | 23 | 19.83 | 1.06 | [0.72-1.56] | 0.766 | 1.19 | [0.84-1.69] | 0.315 |
| Mental illness | No | 935 | 99 | 10.59 | Ref | Ref | Ref | Ref | Ref | Ref |
|  | Yes | 363 | 145 | 39.94 | 3.77 | [3.01-4.72] | <0.001 | 3.01 | [2.41-3.78] | <0.001 |
| Osteoarticular diseases | No | 1079 | 196 | 18.16 | Ref | Ref | Ref | Ref | Ref | Ref |
|  | Yes | 219 | 48 | 21.92 | 1.21 | [0.91-1.60] | 0.195 | 1.05 | [0.79-1.38] | 0.754 |
| Oncological diseases | No | 1237 | 235 | 19.00 | Ref | Ref | Ref | Ref | Ref | Ref |
|  | Yes | 61 | 9 | 14.75 | 0.78 | [0.42-1.44] | 0.408 | 0.70 | [0.42-1.18] | 0.184 |
| Chronic pain | No | 1228 | 229 | 18.65 | Ref | Ref | Ref | Ref | Ref | Ref |
|  | Yes | 70 | 15 | 21.43 | 1.15 | [0.72-1.83] | 0.563 | 0.92 | [0.62-1.35] | 0.663 |

**Mental%=** proportion of the variable within the group referred by mental health; **crudePR=**Crude Prevalence Ratio; **CI95%**=Confidence Interval (95%); **aPR**=Adjusted Prevalence Ratio; **Ref**=Reference category.

**Table 5**. Association between demographic factors, other reasons of referral, chronic diseases and “Functional dependency” as reason of referral to the SP program, Lisbon, Portugal. September 2018 - December 2023 (n=1298).

| **Variables** | **Category** | **Total** | **Functional dependency** | **Funct%** | **crudePR** | **CI95%** | ***p*** | **aPR** | **CI95%** | ***p*** |
| --- | --- | --- | --- | --- | --- | --- | --- | --- | --- | --- |
| **Demographic factors** |  |  |  |  |  |  |  |  |  |  |
| Sex | Female | 841 | 134 | 15.93 | Ref | Ref | Ref | Ref | Ref | Ref |
|  | Male | 457 | 74 | 16.19 | 1.02 | [0.78-1.32] | 0.903 | 1.14 | [0.93-1.41] | 0.212 |
| Age | 0-25 | 154 | 2 | 1.30 | Ref | Ref | Ref | Ref | Ref | Ref |
|  | 26-50 | 353 | 11 | 3.12 | 2.40 | [0.54-10.70] | 0.234 | 2.78 | [0.63-12.33] | 0.179 |
|  | 51-75 | 433 | 42 | 9.70 | 7.47 | [1.83-30.49] | 0.001 | 5.48 | [1.33-22.57] | 0.019 |
|  | >75 | 358 | 153 | 42.74 | 32.91 | [8.26-131.08] | <0.001 | 15.76 | [3.91-63.52] | <0.001 |
| **Other reasons of referral** |  |  |  |  |  |  |  |  |  |  |
| Social and financial support | No | 533 | 98 | 18.39 | Ref | Ref | Ref | Ref | Ref | Ref |
|  | Yes | 765 | 110 | 14.38 | 0.78 | [0.61-1.00] | 0.053 | 0.73 | [0.58-0.92] | 0.008 |
| Social isolation | No | 918 | 148 | 16.12 | Ref | Ref | Ref | Ref | Ref | Ref |
|  | Yes | 380 | 60 | 15.79 | 0.98 | [0.74-1.29] | 0.882 | 0.65 | [0.51-0.84] | 0.001 |
| Sedentary lifestyle | No | 1129 | 197 | 17.45 | Ref | Ref | Ref | Ref | Ref | Ref |
|  | Yes | 169 | 11 | 6.51 | 0.37 | [0.21-0.67] | <0.001 | 0.51 | [0.28-0.91] | 0.024 |
| Mental health | No | 1054 | 188 | 17.84 | Ref | Ref | Ref | Ref | Ref | Ref |
|  | Yes | 244 | 20 | 8.20 | 0.46 | [0.30-0.71] | <0.001 | 0.58 | [0.39-0.88] | 0.011 |
| Unemployment | No | 1041 | 206 | 19.79 | Ref | Ref | Ref | Ref | Ref | Ref |
|  | Yes | 257 | 2 | 0.78 | 0.04 | [0.01-0.16] | <0.001 | 0.13 | [0.03-0.54] | 0.005 |
| **Chronic diseases** |  |  |  |  |  |  |  |  |  |  |
| Cardiovascular diseases | No | 827 | 88 | 10.64 | Ref | Ref | Ref | Ref | Ref | Ref |
|  | Yes | 471 | 120 | 25.48 | 2.39 | [1.86-3.08] | <0.001 | 0.94 | [0.74-1.18] | 0.577 |
| Cerebrovascular disease | No | 1104 | 107 | 9.69 | Ref | Ref | Ref | Ref | Ref | Ref |
|  | Yes | 194 | 101 | 52.06 | 5.37 | [4.29-6.73] | <0.001 | 2.57 | [2.06-3.20] | <0.001 |
| Obesity, overweight, and associated metabolic diseases | No | 1010 | 148 | 14.65 | Ref | Ref | Ref | Ref | Ref | Ref |
|  | Yes | 288 | 60 | 20.83 | 1.42 | [1.09-1.86] | 0.012 | 0.98 | [0.76-1.27] | 0.881 |
| Diabetes | No | 1094 | 151 | 13.80 | Ref | Ref | Ref | Ref | Ref | Ref |
|  | Yes | 204 | 57 | 27.94 | 2.02 | [1.55-2.64] | <0.001 | 1.43 | [1.14-1.79] | 0.002 |
| Respiratory diseases | No | 1182 | 180 | 15.23 | Ref | Ref | Ref | Ref | Ref | Ref |
|  | Yes | 116 | 28 | 24.14 | 1.59 | [1.12-2.25] | 0.013 | 0.84 | [0.59-1.20] | 0.341 |
| Mental illness | No | 935 | 140 | 14.97 | Ref | Ref | Ref | Ref | Ref | Ref |
|  | Yes | 363 | 68 | 18.73 | 1.25 | [0.96-1.63] | 0.097 | 1.26 | [0.99-1.60] | 0.061 |
| Osteoarticular diseases | No | 1079 | 147 | 13.62 | Ref | Ref | Ref | Ref | Ref | Ref |
|  | Yes | 219 | 61 | 27.85 | 2.04 | [1.58-2.65] | <0.001 | 1.41 | [1.11-1.78] | 0.005 |
| Oncological diseases | No | 1237 | 189 | 15.28 | Ref | Ref | Ref | Ref | Ref | Ref |
|  | Yes | 61 | 19 | 31.15 | 2.04 | [1.37-3.03] | 0.001 | 1.53 | [0.98-2.37] | 0.060 |
| Chronic pain | No | 1228 | 188 | 15.31 | Ref | Ref | Ref | Ref | Ref | Ref |
|  | Yes | 70 | 20 | 28.57 | 1.87 | [1.26-2.76] | 0.003 | 1.38 | [0.94-2.01] | 0.097 |

**Funct%=** proportion of the variable within the group referred by functional dependency; **crudePR=**Crude Prevalence Ratio; **CI95%**=Confidence Interval (95%); **aPR**=Adjusted Prevalence Ratio; **Ref**=Reference category.

**Table 6**. Association between demographic factors, other reasons of referral, chronic diseases and “Unemployment” as reason of referral to the SP program, Lisbon, Portugal. September 2018 - December 2023 (n=1298).

| **Variables** | **Category** | **Total** | **Unemployment** | **Unemp%** | **crudePR** | **CI95%** | ***p*** | **aPR** | **CI95%** | ***p*** |
| --- | --- | --- | --- | --- | --- | --- | --- | --- | --- | --- |
| **Demographic factors** |  |  |  |  |  |  |  |  |  |  |
| Sex | Female | 841 | 143 | 17.00 | Ref | Ref | Ref | Ref | Ref | Ref |
|  | Male | 457 | 114 | 24.95 | 1.47 | [1.18-1.82] | 0.001 | 1,38 | [1,14-1,69] | 0,001 |
| Age | 0-25 | 154 | 42 | 27.27 | Ref | Ref | Ref | Ref | Ref | Ref |
|  | 26-50 | 353 | 149 | 42.21 | 1.55 | [1.16-2.06] | 0.001 | 1,51 | [1,12-2,04] | 0,008 |
|  | 51-75 | 433 | 66 | 15.24 | 0.56 | [0.40-0.79] | 0.001 | 0,63 | [0,42-0,94] | 0,024 |
|  | >75 | 358 | 0 | 0.00 | 0.00 | [.-.] | <0.001 | 0,00 | [0,00-0,00] | 0,000 |
| **Other reasons of referral** |  |  |  |  |  |  |  |  |  |  |
| Social and financial support | No | 533 | 94 | 17.64 | Ref | Ref | Ref | Ref | Ref | Ref |
|  | Yes | 765 | 163 | 21.31 | 1.21 | [0.96-1.52] | 0.103 | 0,99 | [0,79-1,25] | 0,931 |
| Social isolation | No | 918 | 215 | 23.42 | Ref | Ref | Ref | Ref | Ref | Ref |
|  | Yes | 380 | 42 | 11.05 | 0.47 | [0.35-0.64] | <0.001 | 0,87 | [0,63-1,19] | 0,374 |
| Sedentary lifestyle | No | 1129 | 233 | 20.64 | Ref | Ref | Ref | Ref | Ref | Ref |
|  | Yes | 169 | 24 | 14.20 | 0.69 | [0.47-1.01] | 0.050 | 0,70 | [0,47-1,06] | 0,089 |
| Mental health | No | 1054 | 203 | 19.26 | Ref | Ref | Ref | Ref | Ref | Ref |
|  | Yes | 244 | 54 | 22.13 | 1.15 | [0.88-1.50] | 0.310 | 1,24 | [0,92-1,67] | 0,158 |
| Functional dependency | No | 1090 | 255 | 23.39 | Ref | Ref | Ref | Ref | Ref | Ref |
|  | Yes | 208 | 2 | 0.96 | 0.04 | [0.01-0.16] | <0.001 | 0,17 | [0,04-0,69] | 0,013 |
| **Chronic diseases** | |  |  |  |  |  |  |  |  |  |
| Cardiovascular diseases | No | 827 | 208 | 25.15 | Ref | Ref | Ref | Ref | Ref | Ref |
|  | Yes | 471 | 49 | 10.40 | 0.41 | [0.31-0.55] | <0.001 | 1,05 | [0,77-1,44] | 0,757 |
| Cerebrovascular disease | No | 1104 | 247 | 22.37 | Ref | Ref | Ref | Ref | Ref | Ref |
|  | Yes | 194 | 10 | 5.15 | 0.23 | [0.12-0.43] | <0.001 | 0,70 | [0,39-1,25] | 0,230 |
| Obesity, overweight, and associated metabolic diseases | No | 1010 | 208 | 20.59 | Ref | Ref | Ref | Ref | Ref | Ref |
|  | Yes | 288 | 49 | 17.01 | 0.83 | [0.62-1.10] | 0.179 | 1,45 | [1,09-1,93] | 0,011 |
| Diabetes | No | 1094 | 231 | 21.12 | Ref | Ref | Ref | Ref | Ref | Ref |
|  | Yes | 204 | 26 | 12.75 | 0.60 | [0.41-0.88] | 0.006 | 0,82 | [0,58-1,16] | 0,256 |
| Respiratory diseases | No | 1182 | 247 | 20.90 | Ref | Ref | Ref | Ref | Ref | Ref |
|  | Yes | 116 | 10 | 8.62 | 0.41 | [0.23-0.75] | 0.002 | 0,64 | [0,35-1,16] | 0,142 |
| Mental illness | No | 935 | 195 | 20.86 | Ref | Ref | Ref | Ref | Ref | Ref |
|  | Yes | 363 | 62 | 17.08 | 0.82 | [0.63-1.06] | 0.125 | 0,93 | [0,70-1,22] | 0,584 |
| Osteoarticular diseases | No | 1079 | 229 | 21.22 | Ref | Ref | Ref | Ref | Ref | Ref |
|  | Yes | 219 | 28 | 12.79 | 0.60 | [0.42-0.87] | 0.004 | 1,19 | [0,86-1,66] | 0,289 |
| Oncological diseases | No | 1237 | 254 | 20.53 | Ref | Ref | Ref | Ref | Ref | Ref |
|  | Yes | 61 | 3 | 4.92 | 0.24 | [0.08-0.73] | 0.003 | 0,41 | [0,14-1,26] | 0,120 |
| Chronic pain | No | 1228 | 246 | 20.03 | Ref | Ref | Ref | Ref | Ref | Ref |
|  | Yes | 70 | 11 | 15.71 | 0.78 | [0.45-1.36] | 0.378 | 1,10 | [0,70-1,72] | 0,688 |

**Unemp%=** proportion of the variable within the group referred by unemployment; **crudePR=**Crude Prevalence Ratio; **CI95%**=Confidence Interval (95%); **aPR**=Adjusted Prevalence Ratio; **Ref**=Reference category.

**Supplementary Material 2.** Characterization of referred patients to the SP program across different referral periods, Lisbon, Portugal. September 2018 - December 2023 (n=1298).

| **Variables** | **Category** | **Before Covid-19 (n=375)** | **During Covid-19 (n=421)** | **After Covid-19 (n=502)** | ***p*** |
| --- | --- | --- | --- | --- | --- |
|  |  | ***n (%)*** | ***n (%)*** | ***n (%)*** |  |
| **Referrals monthly mean (± standard deviation)** | | 10,5 (5,1) | 33,7 (6,6) | 53,2 (5,6) | < 0.001 |
| **Sex** | Female | 253 (67.5) | 269 (63.9) | 319 (63.5) | 0.43 |
|  | Male | 122 (32.5) | 152 (36.1) | 183 (36.5) |  |
| **Age** | 0-25 | 45 (12.0) | 45 (10.7) | 64 (12.7) | 0.011 |
|  | 26-50 | 107 (28.5) | 133 (31.6) | 113 (22.5) |  |
|  | 51-75 | 137 (36.5) | 132 (31.4) | 164 (32.7) |  |
|  | >75 | 86 (22.9) | 111 (26.4) | 161 (32.1) |  |
| **Reasons of referral** | Social and financial support | 187 (49.9) | 287 (68.2) | 291 (58.0) | < 0.001 |
|  | Social Isolation | 140 (37.3) | 99 (23.5) | 141 (28.1) | < 0.001 |
|  | Sedentary lifestyle | 90 (24.0) | 30 (7.1) | 49 (9.8) | 0.001 |
|  | Mental health | 81 (21.6) | 80 (19.0) | 83 (16.5) | 0.16 |
|  | Functional dependency | 35 (9.3) | 95 (22.6) | 78 (15.5) | 0.001 |
|  | Unemployment | 71 (18.9) | 93 (22.1) | 93 (18.5) | 0.35 |
| **Chronic diseases** | Cardiovascular diseases | 110 (29.3) | 146 (34.7) | 215 (42.8) | 0.001 |
|  | Cerebrovascular disease | 45 (12.0) | 65 (15.4) | 84 (16.7) | 0.14 |
|  | Obesity, overweight, and associated metabolic diseases | 63 (16.8) | 92 (21.9) | 133 (26.5) | 0.003 |
|  | Diabetes | 45 (12.0) | 68 (16.2) | 91 (18.1) | 0.046 |
|  | Respiratory diseases | 24 (6.4) | 48 (11.4) | 44 (8.8) | 0.047 |
|  | Mental illness | 111 (29.6) | 115 (27.3) | 137 (27.3) | 0.71 |
|  | Osteoarticular diseases | 53 (14.1) | 74 (17.6) | 92 (18.3) | 0.44 |
|  | Oncological diseases | 18 (4.8) | 24 (5.7) | 19 (3.8) | 0.23 |
|  | Chronic pain | 21 (5.6) | 18 (4.3) | 31 (6.2) | 0.39 |
